# Mycovirus-encoded suppressors of RNA silencing: Possible allies or enemies in the use of RNAi to control fungal disease in crops

**DOI:** 10.3389/ffunb.2022.965781

**Published:** 2022-10-10

**Authors:** Lorena Rodriguez Coy, Kim M. Plummer, Mahmoud E. Khalifa, Robin M. MacDiarmid

**Affiliations:** ^1^ Australian Research Council Research Hub for Sustainable Crop Protection, Department of Animal, Plant and Soil Sciences, La Trobe University, Bundoora, VIC, Australia; ^2^ Botany and Microbiology Department, Faculty of Science, Damietta University, Damietta, Egypt; ^3^ BioProtection, The New Zealand Institute for Plant and Food Research Limited, Auckland, New Zealand; ^4^ School of Biological Sciences, The University of Auckland, Auckland, New Zealand

**Keywords:** mycovirus, dsRNA, RNA interference, suppressor of silencing, HIGS, SIGS

## Abstract

Plants, fungi, and many other eukaryotes have evolved an RNA interference (RNAi) mechanism that is key for regulating gene expression and the control of pathogens. RNAi inhibits gene expression, in a sequence-specific manner, by recognizing and deploying cognate double-stranded RNA (dsRNA) either from endogenous sources (e.g. pre-micro RNAs) or exogenous origin (e.g. viruses, dsRNA, or small interfering RNAs, siRNAs). Recent studies have demonstrated that fungal pathogens can transfer siRNAs into plant cells to suppress host immunity and aid infection, in a mechanism termed cross-kingdom RNAi. New technologies, based on RNAi are being developed for crop protection against insect pests, viruses, and more recently against fungal pathogens. One example, is host-induced gene silencing (HIGS), which is a mechanism whereby transgenic plants are modified to produce siRNAs or dsRNAs targeting key transcripts of plants, or their pathogens or pests. An alternative gene regulation strategy that also co-opts the silencing machinery is spray-induced gene silencing (SIGS), in which dsRNAs or single-stranded RNAs (ssRNAs) are applied to target genes within a pathogen or pest. Fungi also use their RNA silencing machinery against mycoviruses (fungal viruses) and mycoviruses can deploy virus-encoded suppressors of RNAi (myco-VSRs) as a counter-defence. We propose that myco-VSRs may impact new dsRNA-based management methods, resulting in unintended outcomes, including suppression of management by HIGS or SIGS. Despite a large diversity of mycoviruses being discovered using high throughput sequencing, their biology is poorly understood. In particular, the prevalence of mycoviruses and the cellular effect of their encoded VSRs are under-appreciated when considering the deployment of HIGS and SIGS strategies. This review focuses on mycoviruses, their VSR activities in fungi, and the implications for control of pathogenic fungi using RNAi.

## 1 Introduction

Global demand for food and fuel crops has risen over the past few years. At the same time the need for sustainable agriculture has also grown; however, significant abiotic and biotic factors impact plant productivity. Biotic factors include pathogenic microorganisms and invertebrate pests that affect both pre-harvest and post-harvest stages, interfering with both crop yield and quality (Savary et al., 2019; Singh et al., 2022). In the past few decades, alternatives for control of plant pests and pathogens have been sought, because of consumer concerns over environmental and human health, as well as the development of pesticide resistance due to selection pressures arising from decades of over-dependence on agrichemical use for pathogen and pest control (Fisher et al., 2018; Bastos et al., 2021).

Eco-friendly alternatives for crop protection have been attempted with varying degrees of success. These include: biological control using microorganisms, including: *Trichoderma* spp., *Bacillus* spp., *Streptomyces* spp., and *Pseudomonas* spp. (Ferreira and Musumeci, 2021; Pandit et al., 2022); deploying mycoviruses for fungal pathogen control (Gupta et al., 2019); spray treatments using natural plant extracts (Zaker, 2016); and RNA interference (RNAi, also known as RNA silencing) technology that targets pests and pathogens (Liu et al., 2021).

RNAi is a natural process present in many eukaryotes, including plants, where it provides protection against pathogenic microorganisms, with control of plant pathogenic viruses being the most well understood (Ding et al., 2004; Jin et al., 2021). In this process, plants react to the presence of double-stranded RNA (dsRNA) and destroy corresponding RNA sequences (for example viral genomes) using the plant’s silencing machinery (Carbonell and Carrington, 2015). The use of RNAi to control microbes or invertebrate pests depends on the ability of those organisms to take up and process dsRNA, impacting the pest or pathogen by RNAi targeting specific transcripts (Dutta et al., 2015; Qiao et al., 2021). In early examples of RNAi to control microbes, the dsRNA was produced by a transgenic plant and later it was discovered that the dsRNA could be applied directly onto the plant surfaces (Wang and Jin, 2017; Sang and Kim, 2020). Transgenic plants have been a commercial success for controlling various viral diseases (Gonsalves et al., 2004; Rosa et al., 2018). The use of spray applications of dsRNA targeting viruses was an exciting next step to investigate. Targeting invertebrate vectors of viruses came as a natural extension for the use of dsRNA sprays (Kanakala and Ghanim, 2016).

The use of RNAi for fungal disease control was initiated after the discovery of silencing in fungi and the subsequent description of the cross-kingdom RNAi phenomenon. Many fungi have been reported to have RNA silencing machinery (Telengech et al., 2020; Gebremichael et al., 2021) with the most tantalizing recent finding that fungi produce dsRNA to attack their plant host’s defenses (Weiberg et al., 2013). Likewise, plants can counter-attack by delivering dsRNA into pathogenic fungi to target critical transcripts (Cai et al., 2018). This extracellular trafficking of dsRNA, by pathogens and plants, to induce cross-kingdom RNAi inspired the concept of spraying dsRNA onto plants to achieve disease control via the RNAi pathway (Knip et al., 2014; Weiberg et al., 2015). This approach is absolutely dependent on functional RNAi within pathogen cells.

We propose that viruses will impact dsRNA-based management methods resulting in unintended outcomes. Like plants, fungi use RNAi to control fungal viruses (mycoviruses) (Swati et al., 2007; Nuss, 2011). The presence of mycoviruses may disrupt the fungal RNAi and derail the RNAi-based control strategy. This is because viruses, whether plant viruses or mycoviruses, have evolved counter-defence strategies, producing virus-encoded suppressors of RNAi (VSRs), that disrupt their host’s RNAi (Aulia et al., 2021; Ko et al., 2021). Alternatively, the presence of mycoviruses may reduce the fitness of a fungal pathogen and enhance the RNAi-based control. It is therefore necessary to review the roles of mycoviruses, and their VSRs in the context of using RNAi for fungal plant disease control.

## 2 Silencing mechanism in plants and fungi

RNAi is a mechanism that is conserved in plants, fungi, and many other eukaryotes. However, some fungi appear to have lost their RNAi machinery, including species of the subphyla Saccharomycotina, Wallemiomycetes, and some members of the *phylum Microsporidia* (Laurie et al., 2012). The main role of RNAi was discovered to be a defence mechanism against foreign nucleic acids (such as RNA or DNA viruses). It also plays several critical roles in biological processes such as the control of transposon movement, gene regulation, and heterochromatin formation (Ratcliff et al., 1997; Ketting, 2011; Castel and Martienssen, 2013; Gebremichael et al., 2021).

In 1990, an RNAi silencing mechanism was reported (Napoli et al., 1990) for the first time in pigmented petunia flowers; nonetheless, it was not until 1998 that the requirement of dsRNA for gene silencing was demonstrated in *Nicotiana tabacum* (Waterhouse et al., 1998). In filamentous fungi, gene silencing was first reported in the genetic model fungus, *Neurospora crassa*, (Romano and Macino, 1992; Lax et al., 2020).

The RNAi mechanism has been described fully in various reviews (Muhammad et al., 2019; Sang and Kim, 2020; Hung and Slotkin, 2021). In brief, dsRNA is processed by an RNase III, called Dicer (or Dicer-like in plants and fungi), into small interfering RNAs (siRNAs), the siRNAs are incorporated and activated within the RNA-induced silencing complex (RISC) along with the protein Argonaute (AGO or sometimes AGO-like (AGL) in fungi) that targets RNA transcripts through sequence complementarity to the RISC-loaded siRNA. RISC either directs cleavage or inhibition of targeted transcripts. The sequence complementary to the mRNA fragment can also be the primer for RNA-dependent RNA polymerase (RdRp, Rdp, or QDE1 in some fungi) to produce more dsRNAs that initiate and amplify the RNAi cycle again. siRNAs can also be utilized to target methylation of cognate DNA (Cogoni and Macino, 1999). Importantly for this review, RNAi is activated by the presence of dsRNA from either endogenous sources or exogenous origins (Wang et al., 2017).

Although RNAi is a similar process across eukaryotes, there are some variations in the components and their functionality (Abdurakhmonov, 2016). The RNAi machinery in fungi has been associated and reported with multiple functions: antiviral (**Table 1**); control of transposable elements; regulation of endogenous genes; heterochromatin formation; adaptation to stress conditions; and pathogenesis (Lax et al., 2020). As in plants, the number of genes that encode RNAi proteins differs between fungal genera and species (Li et al., 2019; Neupane et al., 2019). The initial mechanism of gene silencing described in *filamentous fungi*, termed quelling, was identified through the introduction of repeated sequences (Romano and Macino, 1992; Cogoni and Macino, 1999). Quelling-defective (qde or QDE1) genes that are required to induce gene silencing in N. crassa were identified and cloned (Cogoni and Macino, 1999). Later, QDE1 was associated as a component of RdRp (Forrest et al., 2004) and both were characterized as nuclear proteins (Nolan et al., 2008). The presence of RdRp within eukaryotic genomes is associated with the evolution of RNA viral genomes carrying an RdRp gene which may have been incorporated into the host genome, but eliminated from the Archaea domain (de Farias et al., 2017). Like the RdRp, termed QDE1, in N. crassa, other RNAi genes may bear alternative names, e.g. fungal AGO proteins are sometimes termed AGO-like (AGL) (Lee et al., 2003; Segers et al., 2006; Yu et al., 2020). The number of genes encoding each protein involved in the RNAi mechanism differs in fungal species and these may be required for distinct cellular activities (Hammond and Keller, 2005; Chen et al., 2015; Son et al., 2017; Yu et al., 2018)

**Table 1:** Fungal RNA interference is an antiviral activity.

Fungus	Evidence, e.g. targeted virus	Reference
*Aspergillus nidulans*	*Aspergillus virus 341*	Hammond et al., 2008
*Botrytis cinerea*	*Botrytis gemydayirivirus 1*	Khalifa and MacDiarmid, 2021
*Colletotrichum higginsianum*	*Colletotrichum higginsianum non-segmented dsRNA virus 1*	Campo et al., 2016
*Cryphonectria parasitica*	*Cryphonectria hypovirus 1*	Sun et al., 2009
*Fusarium graminearum*	*Fusarium graminearum virus 1*	Yu et al., 2020
*Magnaporthe oryzae*	*Pyricularia oryzae ourmiavirus-like virus 1 and 2*	Nguyen et al., 2018
*Neurospora crassa*	*Neurospora crassa fusarivirus 1, Neurospora crassa partitivirus 1*	Honda et al., 2020
*Sclerotinia sclerotiorum*	*Sclerotinia sclerotiorum hypovirus 2-lactuca*	Mochama et al., 2018

## 3 Implications of cross-kingdom RNAi for control of plant pathogenic fungi

Host-induced gene silencing (HIGS) has been deployed commercially for decades to control a range of plant viruses (Gonsalves et al., 2004; de Faria et al., 2016; Rosa et al., 2018) and insects (Baum et al., 2007; Head et al., 2017). HIGS for fungal control has been used only in experimental trials, with siRNA or dsRNA generated by transgenic plants (Nowara et al., 2010; Nunes and Dean, 2012; Raruang et al., 2020; Sang and Kim, 2020; Rajam et al., 2021). To the best of our knowledge, the use of HIGS for fungal control has not as yet been adopted commercially.

Alternatively, siRNA or dsRNA can be applied directly to initiate RNAi to target cognate mRNAs. Various fungi have been shown to take up exogenous dsRNAs from the environment: Fusarium oxysporum and *Mycosphaerella fijiensis* (Mumbanza et al., 2013); *Sclerotinia sclerotiorum* (Regente et al., 2017); *Botrytis cinerea* and *Verticillium dahliae* (Wang et al., 2016). In 2013, Weiberg et al. demonstrated that fungal pathogens secrete siRNAs that are subsequently taken up into plant cells. These fungal origin-siRNAs target host transcripts to suppress host immunity and promote infection. More recently, Jin and colleagues (2021) have demonstrated trafficking of siRNAs between host and pathogen that they termed trans-kingdom or cross-kingdom RNAi (Knip et al., 2014; Weiberg et al., 2015). There are two potential pathways for fungi to take up exogenous dsRNAs or siRNA, i.e., directly whereby the fungal cell takes up RNA that it encounters on host cell surfaces, or indirectly, where the plant first takes up exogenous dsRNA, then processes it into siRNAs, which are then secreted directly or via extracellular vesicles for uptake by the fungal pathogen (Sang and Kim, 2020; Hernández-Soto and Chacón-Cerdas, 2021). The role of vesicles in the delivery of siRNAs has also been demonstrated (Cai et al., 2018). These discoveries led to the proposition that the application of exogenous dsRNA could be used for pest or phytopathogen control (Wang et al., 2016). This dsRNA (or siRNA) introduction may be direct, with spray applications of antifungal dsRNA, in a process known as ‘spray-induced gene silencing (SIGS)’ (Koch et al., 2016; Wang et al., 2017; Qiao et al., 2021).

SIGS has been investigated for control of insect pests (Turner et al., 2006; Zheng et al., 2019), viruses (Mitter et al., 2017; Worrall et al., 2019; Routhu et al., 2022), and fungi (Sang and Kim, 2020; Gebremichael et al., 2021; Niu et al., 2021). Advantages and disadvantages of SIGS and HIGS are discussed elsewhere (Gebremichael et al., 2021; Niu et al., 2021). Importantly, both HIGS and SIGS approaches require a functional RNAi mechanism in the target pest/pathogen for control to be achieved.

Phytopathogenic fungal studies have used HIGS or SIGS to target gene transcripts encoding proteins involved in basic metabolism, such as chitin synthase (Sang and Kim, 2020) or ergosterol biosynthesis (Duanis-Assaf et al., 2022), pathogenicity factors or effectors (McLoughlin et al., 2018). Many SIGS studies (33%) have targeted the fungal RNAi gene silencing mechanisms, e.g., DICER, AGO or vesicle trafficking of siRNAs (Gebremichael et al., 2021). The following HIGS and SIGS studies are not included in the Gebremichael et al., 2021 review and have been used with *Fusarium graminearum* sterol 14α-demethylase genes FgCYP51A, FgCYP51B and the virulence factor FgCYP51C, in Arabidopsis and barley (Koch et al., 2019), and the Fg00677, Fg08731 (two essential protein kinases), and CYP51 (cytochrome P450 lanosterol C14-α-demethylase) genes in *Brachypodium distachyon* (He et al., 2019). HIGS has also been used to target *Sclerotinia sclerotiorum* genes Ssoah1 (regulatory pivot for oxalic acid production) (Rana et al., 2022), Ss-caF1 (putative Ca2+ binding protein), SspG1d (endopolygalacturonase), and SsiTL (integrin) (Maximiano et al., 2022) and when combatting *Phakopsora pachyrhizi*, three genes (ATC: *acetyl‐CoA acyltransferase*, GCS_H: *glycine cleavage system H protein*, and RP_S16: 40S ribosomal protein S16) of eight genes evaluated, reduced the number of pustules of *P. pachyrhizi* (Hu et al., 2020).

## 4 Mycoviruses and their encoded suppressors of RNA silencing

Mycoviruses (viruses that multiply within fungi) and several plant viruses, are hosted and vectored by members of the fungal kingdom (Jiang et al., 2013; Buck, 2018; Bian et al., 2020). With over 6 million species, the fungal kingdom comprises microorganisms with varying morphologies, life cycle strategies, and economic significance (Taylor et al., 2014). Mycoviruses have been reported in members of the three major fungal phyla: *Chytridiomycota*, *Ascomycota*, and *Basidiomycota* (Pearson et al., 2009). Note that the *Zygomycota* are no longer recognized as Fungi (Spatafora et al., 2017; Tedersoo et al., 2018). Fungi are extensively infected with mycoviruses (Ghabrial, 1998; Hong et al., 1999), which are hosted in their cytoplasm or limited to the mitochondria (Polashock et al., 1997; Varga et al., 2003). Although the antiviral defences of the host fungi may limit the challenge of infection by some mycoviruses, successful mycovirus infections can have an observed impact on their fungal host’s growth and even impact pathogenicity of some phytopathogenic fungi. Mycovirus-encoded counter defences that reduce the RNAi activity of a target fungus could also decrease the efficacy of SIGS and HIGS due to the requirement of this activity for silencing to function.

### 4.1 The form and taxonomy of mycoviruses

Currently, three genome types, double stranded RNA (dsRNA), single-stranded RNA (ssRNA), and single-stranded DNA (ssDNA) are reported and verified as mycoviral genomes, with dsRNA historically being the most often reported among all known mycoviruses (Kondo et al., 2022). This dsRNA genome bias may be related to the ‘pre-sequencing era’ use of dsRNA purification and profiling to detect the presence of fungal viruses. Unlike most ssRNA mycoviruses, most dsRNA viruses are encapsidated. Mycoviruses have been categorized into at least 27 viral families with certain genera remaining unclassified and unassigned to any known virus family (Mu et al., 2021; Ruiz-Padilla et al., 2021; Myers and James, 2022; Kondo et al., 2022, Matthijnssens et al. in press). To date, mycoviruses have been recorded (established or proposed) to include: members within dsRNA virus families (*Amalgaviridae, Chrysoviridae, Curvulaviridae, Megabirnaviridae, Partitiviridae, Polymycoviridae, Quadriviridae, Spinareoviridae* (previously *Reoviridae*), *Totiviridae and Botybirnaviridae*); members within positive sense ssRNA virus families (*Alphaflexiviridae, Barnaviridae, Botourmiaviridae, Deltaflexiviridae, Endoraviridae, Fusariviridae, Gammaflexiviridae, Hadakaviridae, Hypoviridae, Mitoviridae, Narnaviridae, Yadokariviridae*); members within positive sense ssRNA families that act like retroviruses (*Metaviridae and Pseudoviridae*); members within negative-sense ssRNA virus families (*Mymonaviridae and Phenuiviridae*); and *Genomoviridae*, which comprises circular ssDNA viruses and represents the sole DNA mycovirus family as no dsDNA mycoviruses have been described as yet (Hisano et al., 2018; Khan et al., 2021; Kondo et al., 2022; Matthijnssens et al., 2022 in press; Walker et al., 2020).

### 4.2 Technologies used to discover mycoviruses

The rate of mycovirus discovery has surged over the past decade due to the application of semi- and non-targeted high throughput sequencing (HTS). Previously, mycoviruses were detected based on the presence of dsRNA in total RNA or dsRNA-enriched extracts from fungi, then the genome could be identified by sequencing cloned genomic pieces (Khalifa et al., 2016). With the development of HTS methods, mycovirus discovery has become increasingly practical, and numerous mycovirus sequences are being uploaded to the GenBank databases on a continuous basis (Ho and Tzanetakis, 2014). A range of HTS methods are used based on total RNA (sometimes ribo-depleted) or total DNA (fungal genome including integrated or non-integrated viruses), or purified virions or virus enriched nucleic acid structures, e.g. dsRNA from RNA and/or DNA viruses or circular DNA multiplied by rolling circle amplification (Fitzpatrick et al., 2021). However, knowledge of mycovirus cellular biology, host interaction, and environmental impacts is still limited.

Pioneer virologists focused solely on hosts displaying symptoms to discover associated viruses, whereas today’s virologists can target any host or even environmental samples more easily, using high throughput sequencing technologies. These technologies enable discovery of viruses within their host (enabling immediate association with at least one host) or in the absence of their host (e.g., in water or associated with top predator insects such as dragonflies). The former approach requires only confirmation of the host using a second method, whereas the latter approach requires ‘de-orphaning’ of the virus sequence to identify the host(s) in which the genome replicates. Both approaches are agnostic to symptoms that may be associated with virus infection. Subsequent biological studies of the virus-host interaction are required to understand the impacts of the virus-host interactions within different abiotic and biotic conditions. The focus on fungi as hosts of viruses intensified from 2012 to the present day when the application of HTS increased the rate of mycovirus discovery (Mokili et al., 2012; Rosario et al., 2012; Khalifa et al., 2016). Therefore, mycovirology, founded 60 years ago with the discovery of the first mycoviruses (Hollings, 1962), is an emerging discipline that is now primed with methods that enable the rapid discovery of new species, genera, and families but requires essential biological research to understand mycovirus impacts in their hosts and the wider environment.

### 4.3 Mycovirus prevalence in fungi

The prevalence of mycoviruses in filamentous fungi ranges from all to no isolates infected with one or more mycoviruses depending on the host/virus combination, research location, as well as how the mycovirus research was conducted (**Table 1S**, Pearson et al., 2009; Ghabrial et al., 2015). To our knowledge, only six studies have quantified directly (with no sub-culturing step) the natural prevalence of mycovirus infection using different methods to detect mycovirus infection. These studies have detected upwards of 19% prevalence. Recently, 20 partitiviruses with representatives across three genera were characterized by Sanger sequencing of viral dsRNAs and HTS on total RNAs from 16 field isolates of *Rosellinia necatrix* (125% incidence, though these may have been pre-screened for dsRNA presence, Telengech et al., 2020). By contrast, dsRNA bands were detected from only 21% of field isolates of *R. necatrix* (Arakawa et al., 2002). All 248 field isolates of Botrytis cinerea isolated from grapevines were mycovirus infected compared with cultured isolates that had lower prevalence (Ruiz-Padilla et al., 2021, **Table 1S**). High prevalence (100–34%) of Ustilago maydis virus H1 (Umv-H1) was detected in field isolates of Ustilago maydis from maize and teosinte growing in USA and Mexico (Voth et al., 2006). When studying the causative agent of Phoma stem canker disease on oilseed rape 69% of the isolates of Leptosphaeria biglobosa were mycovirus infected, but none of the L. Maculans isolates (that cause black leg on rape) had detectable mycovirus infections (Shah et al., 2020). In *Magnaporthe oryzae*, the pathogen that causes rice blast, and arguably the most important plant pathogen in the world, 11 of 58 isolates carried dsRNA elements (Urayama et al., 2010). By contrast to mycovirus prevalence in field isolates, only two of 105 isolates of cultured Fusarium species were found to be infected with mycoviruses in a recent investigation that sequenced isolated dsRNA (Jacquat et al., 2020). A wide range of mycovirus prevalence (100–2.2%) has been detected in Botrytis cinerea by different methods though no direct methodological comparison was investigated (**Table 1S**, and references therein). While different detection technologies ranging from metatranscriptomics, virus-specific amplification, enrichment by virion-associated nucleic acid (Filloux et al., 2015) or rolling circle amplification (James et al., 2011) or dsRNA have been applied, the most common method used to estimate the prevalence of mycoviruses has been traditional dsRNA band profiling. Viruses have also been identified, using a range of similar techniques, in Oomycetes (Botella and Jung, 2021; Fukunishi et al., 2021; Raco et al., 2022); however, as Oomycetes are not fungi, these have not been included in this review.

Several studies have shown that the reported prevalence of mycovirus infection may not be accurate because cultured isolates were used thereby providing opportunity for the elimination of mycoviruses that are lost through passaging (Bao and Roossinck, 2013). Further inaccuracies in reporting may arise through use of dsRNA bands as a proxy for mycovirus infection. Banding profiles of dsRNA do not consistently correlate with specific virus infections (Raco et al., 2022). Some single-stranded RNA viruses do not seem to accumulate dsRNA in infected mycelium and therefore are under-represented by dsRNA band profiling. Similarly, DNA mycoviruses are not accounted for using dsRNA profiling and were not discovered within fungi until this century when rolling circle amplification was applied to enrich for these circular structures (Herrero et al., 2009). Another virus discovery method VANA enriches virion-like particles but this method disregards unencapsulated mycoviruses (Chong et al., 2018; Hall et al., 2014). Perhaps the method that most faithfully discovers all mycoviral structural forms is metatranscriptomics, which compensates an enrichment step through high sequence depth of all RNA transcripts present in a cell, then relies on bioinformatic analyses to discriminate host transcripts from those copied from either RNA or DNA mycoviruses. For instance, the recent non-targeted metatranscriptome survey by Ruiz-Padilla and colleagues (2021) revealed that the majority of 248 B. cinerea grapevine isolates from Italy and Spain carried one or more mycoviruses, including some mycoviruses not previously associated with B. cinerea and four bisegmented viruses (binarnaviruses) that were new to science. High-throughput sequencing of transcriptomes (RNAseq) from ericoid and orchid mycorrhizal fungi identified viruses belonging to already known taxa as well as previously uncharacterized members (Sutela et al., 2020). In another study conducted to monitor the interannual dynamics and abundance of mycoviruses infecting S. sclerotiorum within a rapeseed-field, 68 mycoviruses were identified, among which 28 were novel (Jia et al., 2021).

### 4.4 The impact of mycovirus infection on the pathogenicity of their host fungus

Mycoviruses are associated with the full spectrum of impacts on their phytopathogenic fungal hosts; hypovirulence, through latency and/or seemingly benign impacts, to hypervirulence (Kotta-Loizou, 2021). The impact(s) of mycovirus infections in non-pathogenic fungi is unknown. Mycoviruses hijack the intracellular processes to result in changes in the host transcript and protein expression and functioning, in part due to manipulation of the antiviral RNA silencing mechanism (Myers and James, 2022). Hypovirulence describes when the host phytopathogenic fungus has no or very low pathogenicity when infected with a mycovirus. An example is Cryphonectria hypovirus 1 (CHV1) that reduces the phytopathogenicity of infected *Cryphonectria parasitica* thereby providing a unique form of biological control for the chestnut blight pathogen (Shapira et al., 1991). Latent mycovirus infection is where there is no apparent change to the fungal host when infected with a mycovirus, as exampled by most mycoviruses that are members of Totiviridae (Yie et al., 2014; Khalifa and MacDiarmid, 2019), Chrysoviridae (Kotta-Loizou et al., 2020) or Endornaviridae (Valverde et al., 2019). Other examples of mycovirus infection cause changes in sporulation or sectoring patterns on culture plates (Ejmal et al., 2018a, and Ejmal et al., 2018b). Other mycovirus infections can be observed as causing benefits to the immediate and/or secondary host, such as thermal tolerance (Márquez et al., 2007). Finally, hypervirulence is when the mycovirus-infected fungus is more pathogenic or has heightened pathogenicity compared to the uninfected fungus, as recently exampled by a potential member of the Partitiviridae and Rhabdoviridiae (Olivé and Campo, 2021; Li et al., 2022). Further research to describe the role/s of mycoviruses within pathogenic (and non-pathogenic) fungi and to directly test their impacts on their host(s) is necessary to elucidate the full influence of mycoviruses in various ecosystems. Finally, understanding the mechanism(s) by which mycoviruses influence their host (and secondary hosts) will enable their manipulation and use to maximise their potential in reducing pathogen burdens and response to a changing climate.

### 4.5 Mycovirus-encoded suppressors of RNA silencing (myco-VSRs)

One of the tactics used by viruses to fight host antiviral machinery is to encode one or more VSRs; for mycovirus we use the term myco-VSRs. Typically, VSRs reduce the RNAi activity within their host to enable virus genome replication, sub-genomic RNA production and movement of the virus into new cells (Yang and Li, 2018). By altering the normal functioning of RNAi in the cell, the VSR may perturb the gene regulation required for development and cross-kingdom communication that typically occurs in an uninfected cell. Gene transcripts that are typically downregulated by micro-RNAs may become upregulated and siRNAs that are produced for defence or pathogenicity may be reduced in number, sequestered, or otherwise rendered ineffective. Such suppression of RNAi may be one of the mechanisms by which mycovirus-associated hypovirulence is conferred to an otherwise pathogenic fungus (**Table 2**). Myco-VSRs present in target fungal cells are also likely to negatively impact the efficacy of SIGS and HIGs as these both require functional RNAi in the target cell. Identification of myco-VSRs and knowledge of their mode of action will assist in developing efficacious pathogenic fungal control methods.

**Table 2:** Mechanisms of action used by mycovirus-encoded suppressors of RNA silencing (myco-VSRs) to repress fungal RNAi.

Fungus	Virus	Myco-VSR	Myco-VSR protein function	Mechanism	Reference
*Aspergillus nidulans*	*Aspergillus virus 1816*	–	–	Reduction of small interfering RNA accumulation	Hammond et al., 2008
*Cryphonectria parasitica*	*Cryphonectria hypovirus 1*	p29	Papain-like cysteine protease symptom inducer RNA silencing suppressor	Reduction in transcription level of DCL2 and AGL2	Segers et al., 2006; Segers et al., 2007
	*Cryphonectria hypovirus 4*	P24	A protease antiviral RNA silencing suppressor		Aulia et al., 2021
*Fusarium graminearum*	*Fusarium graminearum virus 1*	P2	–	Suppresses transcriptional upregulation of the key enzymes genes, i.e., FgDICER2 (a dcl) and FgAGO1 (an agl or ago)	Yu et al., 2020
*Rosellinia necatrix*	*Rosellinia necatrix mycoreovirus 3*	P10 or S10	–	Not determined	Yaegashi et al., 2013
*Orchid mycorrhizal fungi (Tulasnella)*	*Tulasnella partitivirus 2* *Tulasnella partitivirus 3*	CP	Structural protein RNA silencing suppressor		Shimura et al., 2022

To date, four myco-VSRs have been described and one was inferred (**Table 2**). The first myco-VSR described was for p29 encoded by *Cryphonectria hypovirus* 1 (CHV1-EP713) by Segers and colleagues who used both the natural Cryphonectria parasitica host or a plant host overexpressing a hairpin GFP construct to detect VSR activity (Segers et al., 2006; and Segers et al., 2007). Zhang and colleagues (2008) described the specific repression of the host C. parasitica dcl-2 in a manner dependent on expression of p29 papain-like protease encoded by Cryphonectria hypovirus 1 (CHV1-EP713). Aulia and colleagues (2021) demonstrated myco-VSR activity of the Cryphonectria hypovirus 4 (CHV4) encoded p24, a homolog of CHV1 p29. It is likely that these myco-VSR activities of both CHV1 and CHV4 support the co-infection of mycoreovirus 1-Cp9B21 (MyRV1-Cp9B21) and mycoreovirus 2 (MyRV2), respectively, in C. parasitica resulting in additional symptoms or sustained infections compared with single mycoreovirus infections (Sun et al., 2006; Aulia et al., 2019). By contrast, using GFP expressed from either the F. graminearum DCL2 or AGO1 promoter in the presence of Fusarium graminearum virus 1 (FgV1) or assessing host transcript accumulation in the presence of each ectopically expressed FgV1 coding region, pORF2 was identified as a VSR that targets the promoter regions of FgDICER2 and FgAGO1 (Yu et al., 2020). Using a reporter and dsRNA inducer method, a *Rosellinia necatrix* line overexpressing GFP and a dsRNA GFP was tested against four divergent mycoviruses to determine whether they could suppress the silencing of the GFP. Only the mycoreovirus (Rosellinia necatrix mycoreovirus 3; RnMyRV3) showed VSR activity and the RnMyRV3 p10 (S10) suppressed RNAi in the widely used *Nicotiana benthamiana* 16c line, likely by interfering with siRNA production as these accumulated to only low levels (Yaegashi et al., 2013). Likewise, VSR activity was determined by Hammond and colleagues (2008) who induced RNAi targeting an mRNA required for synthesis of a secondary metabolite in *Aspergillus* species then monitored the metabolite in the presence or absence of mycoviruses. From the three different mycoviruses tested, only *Aspergillus virus* 1816 within A. nidulans was capable of suppressing RNAi and this resulted in reduced siRNA. It has also been demonstrated that a fungal host’s RNAi machinery is upregulated in the presence of mycovirus that lacks a VSR (compared to one that has an active VSR) (Hammond et al., 2008; Zhang et al., 2008).

Determining the presence and prevalence of mycovirus-encoded suppressors by bioinformatics is often impractical since there are no generalized, conserved sequence motifs characteristic for VSRs across different virus groups/families. Inference of myco-VSR presence can only be made between related viruses, where there may be some remaining sequence similarity. Commonly, empirical methods are used to determine VSR activity associated with each virus-encoded protein. Standard techniques used to date have predominantly been for plant-VSRs (Roth et al., 2004), or using plant-based methods to identify suppressors of RNAi encoded by entities other than plant viruses, e.g., the plant host itself (rgs-CaM protein) (Anandalakshmi et al., 2000), human- or insect-VSRs (Li et al., 2004). Other methods include overexpression of the potential VSR in the fungal host (wildtype or mutant for a gene whose encoded protein is involved in the RNA silencing machinery) and detection of changes in RNAi metabolism compared to cells lacking VSR expression (Aulia et al., 2021, **Table 2**).

## 5 Scenarios of SIGS in the presence of mycoviruses in fungi

**Figure 1:**
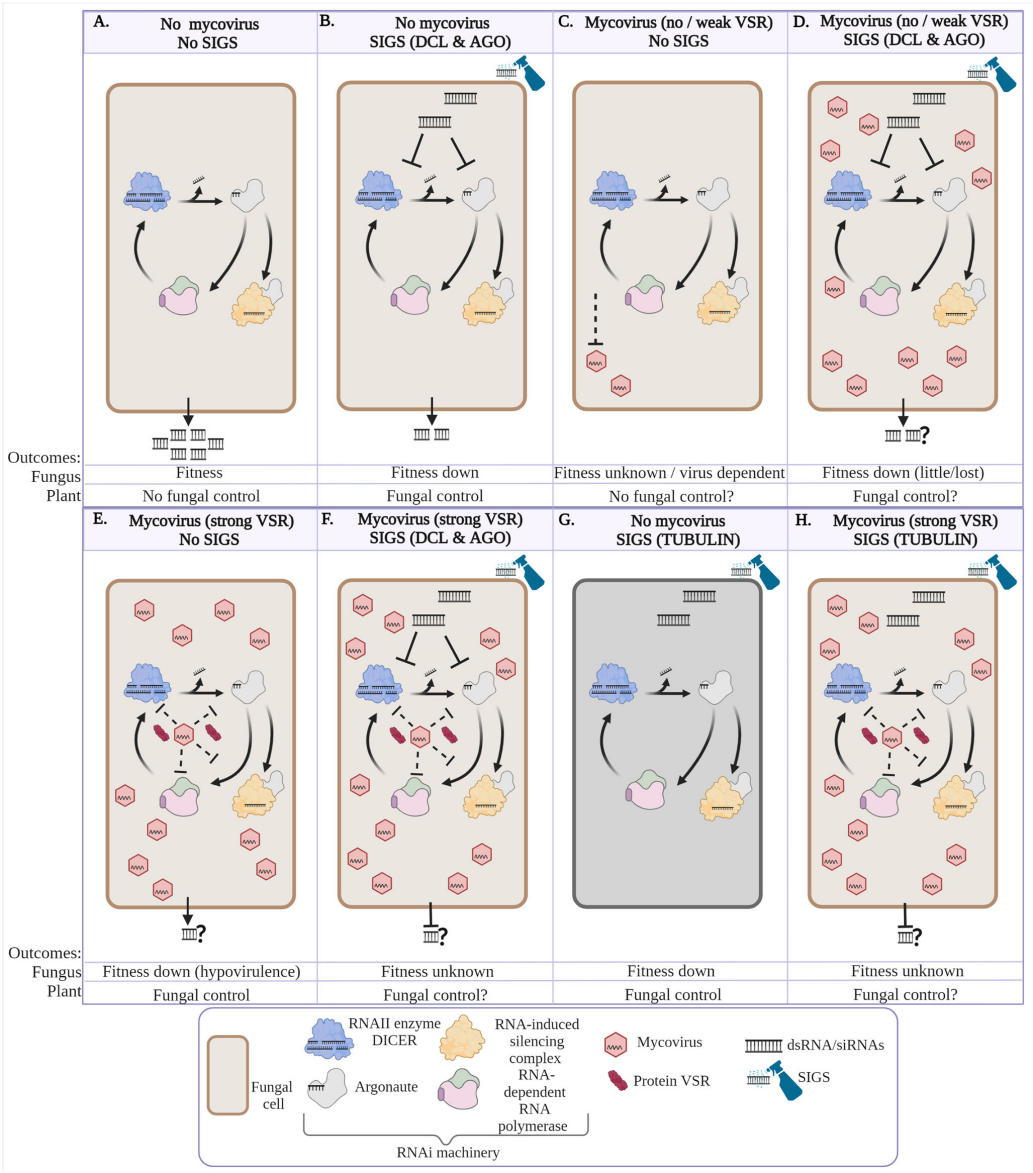
Various scenarios depicting the impact of fungal control using spray-induced gene silencing (SIGS) targeting fungal silencing machinery and mycovirus infection. These scenarios are particularly relevant for those fungi that produce dsRNA to target the host defence response. **(A)** Fungus produces either double stranded RNA (dsRNAs) and/or small interfering RNAs (siRNAs) to suppress host plant immunity or regulate virulence genes (effectors). **(B)** The dsRNAs and/or siRNAs from SIGS are taken up by a fungus and processed by RNAIII enzyme, DICER. Then, Argonaute (AGO) and RNA-induced silencing complex (RISC) include one siRNA strand that guides silencing of DCL1/2 and AGO1/2 messenger mRNA (mRNA). Alternatively, siRNAs prime RNA-dependent RNA polymerase (RdRP) to create more dsRNA that amplifies the RNAi machinery to target more DCL1/2 and AGO1/2 mRNAs. In scenario B the RNAi activity in the cell will be reduced resulting in few siRNA required for pathogenesis. If the fungus relies on siRNAs as key secreted virulence factors, then fungal fitness and pathogenicity will be reduced. **(C)** Fungus is infected by mycovirus with no or weak virus-encoded suppressors of RNAi (VSRs) resulting in mycovirus replication that is limited by active fungal host RNAi (dependent on specific mycovirus and fungal host interaction). **(D)** This scenario depicts the combinations of A and B, where the fungus has a mycovirus and SIGS targets either DICER or AGO resulting in reduced antiviral activity, increased mycovirus replication and reducing fungal virulence compared with scenario **(C)** virulence. **(E)** Mycovirus produces VSRs to deactivate the fungal RNAi machinery (DICER, AGO, RdRP or other RNAi targets/activities), resulting in mycovirus accumulation and reduction in fungal fitness. **(F)** Depicts a scenario of a fungus with SIGS (targeting DICER and AGO) and a mycovirus encoding strong VSR activity; the outcome is unknown, and probably dependent on a balance between mycovirus, VSR activity and strength, and the efficiency of the dsRNA to achieve silencing. **(G)** Depicts a fungus with SIGS applied to target β-tubulin (a protein not involved in RNAi). Fungal death results from the inhibition of tubulin polymerization. **(H)** depicts a scenario that combines E and G whereby the fungal cell is infected with a mycovirus with strong VSR activity and tubulin polymerization is inhibited via RNAi. Fitness of the fungus in this scenario will depend on the balance between SIGS-induced RNAi efficacy and VSR deactivation of the RNAi machinery similar to scenario **(F)**. The illustration was created with BioRender.

Mycoviruses have the potential to impact the outcome when HIGS or SIGS is deployed for phytopathogenic fungal control. Here we discuss these potential impacts using SIGS as an example; however, the same issues are relevant to HIGS. **Figure 1A–H** represents our predicted scenarios of interactions between fungi, with and without mycoviruses (that in turn may, or may not, encode VSRs), in the absence or presence of deployed SIGS. A SIGS-treated fungus is depicted either targeting the RNAi machinery (DCL and AGO, **Figures 1B, D, F**) or β-tubulin (**Figures 1G, H**). These scenarios are particularly relevant for those fungi that produce dsRNA to target the host defence response. **Figures 1E, F, H** show the scenarios with SIGS in the presence of mycoviruses which encode a VSR that may alter the outcome of SIGS.

Fig 1A illustrates a fungus producing and delivering ds/siRNAs to suppress host plant immunity, a key function in fungal phytopathogenicity (Weiberg and Jin, 2015; Cai et al., 2018). **Figure 1B** represents SIGS targeting the silencing mechanism (DCL1/2 and AGO1/2) of a fungus that is not infected by a mycovirus. Here, the SIGS-delivered dsRNA initiates silencing of the target transcripts DCL1/2 or AGO1/2. As the DCL1/2 or AGO1/2 transcript targets decrease in abundance, and the pre-existing DCL1/2 or AGO1/2 proteins expire, the fungal RNAi machinery reduces in efficacy with negative impacts on general fungal metabolism and phytopathogenicity. With no (or reduced) silencing machinery, the fungus is unable to produce and deliver dsRNA into the plant to suppress plant defences thereby enabling the plant’s defences to control the fungal infection (Wang et al., 2016). The fungal fitness is reduced, and the fungus is controlled at least temporarily. Over time the SIGS becomes less effective due to dependence on RNAi activity (including DCL1/2 or AGO1/2) to cleave their own target DCL1/2 or AGO1/2 transcripts.

Despite the prevalence of mycoviruses of up to 100% of all fungal isolates examined (Voth et al., 2006), the interaction between a fungus and a mycovirus has not been previously considered or studied in the context of SIGS or HIGS. To the best of our knowledge, no reported studies that utilize HIGS or SIGS to alter or examine fungal-plant interactions have determined the mycoviral status of the experimental fungal strains.

**Figure 1C** represents the scenario where a fungus infected with a mycovirus (either encoding no, or a weak VSR) is able to limit the mycoviral infection through fungal silencing machinery. In this scenario, the mycovirus does not accumulate (or to only a limited extent) and the fungus remains relatively fit and able to infect the plant. This scenario could represent latent mycovirus infections of a fungus. Similarly, **Figure 1D** represents the scenario described in **Figure 1C** with the additional application of SIGS targeting the silencing mechanism (DCL1/2 and AGO1/2). In this scenario, if SIGS effectively reduces fungal RNAi activity, uncontrolled mycovirus replication may occur, thereby debilitating the fitness and the pathogenicity of the fungus. A scenario similar to this has been demonstrated in an elegant study by Mochama et al., 2018; however, in their study gene knockouts were used to remove silencing machinery, rather than SIGS. In this study, the authors showed that a Δdcl-1/dcl-2 double knockout mutant in Sclerotinia sclerotiorum grew more slowly compared with wildtype; however, infection by the fungus of its host plant was not affected. When the knockout mutant fungus was infected with a mycovirus its in vitro growth and infection of the plant were severely affected (Mochama et al., 2018). This study has significant relevance for the proposal of using HIGS or SIGS in the field. For this reason it recommended that HIGS and SIGS studies compare fungal strains with and without a range of mycoviruses in different fungal host species.

Mycoviruses can suppress the fungal RNAi silencing machinery thereby enabling a mycovirus to replicate and thrive in the fungus. The mycovirus VSR(s) may suppress either DCL or AGO (**Table 2**), or potentially RdRp, siRNA movement or other RNAi activities (**Figure 1E**). It is important therefore to consider the role of mycoviruses and their encoded VSRs for the control of fungi using SIGS or HIGS. **Figure 1F** represents the scenario described in **Figure 1E** but in the presence of SIGS targeting DCL1/2 and AGO1/2. If SIGS were applied under these circumstances, the dsRNA or siRNA taken up by the fungus may be inactivated (as observed by some plant-VSRs) via direct binding by the myco-VSR or indirectly by the VSR inactivating the DCL, AGO or RdRp (reviewed in Rahman et al., 2021). Due to the lack of research, it is unknown what the outcome would be and whether all mycoviruses (and their encoded VSRs) may have a similar or distinct impact. Such an interaction may result in no impact to the mycovirus-infected and SIGS-treated fungus (resulting in the same outcome as depicted in **Figure 1E**), or SIGS applied dsRNA or siRNA may outcompete stoichiometrically, the myco-VSR(s) (resulting in the same outcome as depicted in **Figure 1B**) or may result in uncontrolled mycovirus replication as described and depicted in **Figure 1D**.

Inhibitors of tubulin polymerization are commonly used to control phytopathogens (Young, 2015). **Figure 1G** illustrates the scenario where the β-tubulin transcript is targeted by SIGS, resulting in disruption of cell division leading to fungal cell death. **Figure 1H** represents an alternative scenario that is similar to **Figure 1F**, except that the SIGS target is not a transcript encoding a component of the fungal RNAi machinery, but instead a protein necessary for basic metabolism, such as β-tubulin, as described and depicted in **Figure 1G**. In such a scenario, the SIGS RNAi may not directly affect the fungal-mycovirus (and its VSR) balance. However, the SIGS would remain dependent on effective RNAi activity in the fungal cells. The outcome would likely depend on the cellular target and strength and activity of any myco-VSR(s), if present, and how efficiently they suppress the SIGS-initiated RNAi targeting of β-tubulin. 

## 6 Future research

It is clear that additional research is required to determine the impact of mycoviruses on HIGS or/and SIGS for control of fungal plant pathogens. In particular, the presence or absence of mycoviruses, the role of VSRs, and their interactions with HIGS and SIGS should be demonstrated with a range of fungal plant pathogens and mycoviruses. To aid discovery, a rapid and standard method to identify the presence of myco-VSR activity, including strength and mechanism of action would be of great benefit to the mycovirus, fungal biology, and wider biology community. The implication of mycovirus infection in a fungal field population context should also be taken into consideration prior to deploying HIGS or SIGS in the field. As described in **Figure 1C**, some mycoviruses will benefit from the application of HIGS or SIGS. The potential for selecting members of the fungal population through application of dsRNA or siRNAs is a risk, which needs to be determined.

## 7 Conclusions

The development of novel and environmentally sustainable control options for plant pathogenic fungi is critical for future crop production. A greater understanding of the role of mycoviruses in fungal pathogen systems will enable us to harness the potential of HIGS and/or SIGS for fungal disease control.

## Author contributions

All authors wrote the first draft of the manuscript, then edited and reviewed the manuscript prior to submission.

## Funding

The research is financially supported by the La Trobe University Post Graduate Research Scholarship, La Trobe University Full Fee Research Scholarship, by the Australian search Council Research Hub for Sustainable Crop Protection (project number IH190100022) founded by the Australian Government, and Growing Futures™ programme (Science Services Investment Fund) of The New Zealand Institute for Plant and Food Research Limited.

## Acknowledgments

The authors would like to acknowledge Drs Kieren, Arthur, Joanna Bowen, Anthony Gendall and Scott Mattner forreviewing the manuscript prior to submission. The research is financially supported by the La Trobe University Post Graduate Research Scholarship, La Trobe University Full Fee Research Scholarship, by the Australian Research Council Research Hub for Sustainable Crop Protection (project number IH190100022) founded by the Australian Government.

## Conflict of interest

The authors declare that the research was conducted in the absence of any commercial or financial relationships that could be construed as a potential conflict of interest.

## Publisher’s note

All claims expressed in this article are solely those of the authors and do not necessarily represent those of their affiliated organizations, or those of the publisher, the editors and the reviewers. Any product that may be evaluated in this article, or claim that may be made by its manufacturer, is not guaranteed or endorsed by the publisher.
