# Supplementary material for: Mycovirus-encoded suppressors of RNA silencing: Possible allies or enemies in the use of RNAi to control fungal disease in crops
Source: Front Fungal Biol. 2022 Oct 10;3:965781. doi: 10.3389/ffunb.2022.965781 (PMC10512228; doi:10.3389/ffunb.2022.965781)
Supplement: Supplementary file 1 [file Table_1.docx]

Supplementary Material

## Table 1S. Mycovirus prevalence in isolates of fungal species reported in published studies across the world using different discovery methods.

| **Mycovirus prevalence** | **Mycovirus detection method** | **Host fungus** | **Fungus- associated disease** | **Fungus isolate (country, host of fungus [as available], field/cultured)** | **Reference** |
| --- | --- | --- | --- | --- | --- |
| 125% | High throughput sequencing pre-screened for dsRNA | Rosellinia necatrix | White root rot | Japan and Spain, unnamed perennial crops, field | Telengech et al., 2020 |
| 21% | dsRNA bands |  |  | Japan, 27 (unnamed) plant species and soil, field | Arakawa et al., 2002 |
| 100-34% | dsRNA bands | *Ustilago maydis* | Corn smut | United States and Mexico, *Zea mays* and Zea (teosinte), field | Voth et al., 2006 |
| 100% | Metatranscriptomics | *Botrytis cinerea* | Grey mold | Italy and Spain, *Vitis vinifera*, field | Ruiz-Padilla et al., 2021 |
| 72% | dsRNA bands |  |  | New Zealand, *Cucumis sativus*, *V. vinifera*, *Solanum lycopersicum*, *Fragaria* × ananassa, *Phaseolus vulgaris*, *Rubus fruticosus*, cultured | Howitt et al., 1995 |
| 29% | Botrytis virus X RT-PCR |  |  | New Zealand, a wide range of plants, cultured | Pearson and Bailey, 2013 |
| 16% | Botrytis virus F RT-PCR |  |  | International, a wide range of plants, cultured | Pearson and Bailey, 2013 |
| 2% | Rolling-circle amplification and high throughput sequencing |  |  | New Zealand, a wide range of asymptomatic plants, cultured | Khalifa and MacDiarmid 2021 |
| 84% | dsRNA bands | *Chalara elegans* synonym *Thielaviopsis basicola* | Black root rot | United States, Canada, New Zealand, and the Netherlands, *Gossypium* spp., *Cyclamen* spp., *Daucus carota*, *Nicotiana tabacum*, *Vinca spp*., *Petunia* *spp*., *Cicer arietinum*, *Leonurus cardiaca*, cultured | Bottacin, et al, 1994 |
| 78% | dsRNA bands | *Beauveria bassiana* | Entomopathogenic fungus that causes white muscardine | New Zealand, *Declana floccosa*, *Aenetus virescens*, *Vespula germanica*, *Hyperodes bonariensis*, *Vespula germanic*, *Hylastes ater,* cultured | Yie et al., 2014 |
| 74% | dsRNA bands | *Monilinia fructicola* | Brown rot | New Zealand, *Prunus persica* and *P. persica* var. nucipersica, cultured | Pi-Fang et al., 2004 |
| 69% | dsRNA bands | *Leptosphaeria biglobosa* | Phoma stem canker | China, *Brassica napus*, field | Shah et al., 2020 |
| 68% | dsRNA bands | *Helicobasidium mompa* | Violet root rot | Japan, *Malus domestica*, *Morus alba*, *Ipomoea batatas*, and other plants, or land, cultured | Ikeda et al., 2004 |
| 28% | dsRNA bands | *Cryphonectria parasitica* | Chestnut blight | North America, *Castanea dentate*, cultured | Peever et al., 1997 |
| 12% | dsRNA bands |  |  | Korea, *Castanea crenata*, cultured | Park et al., 2008 |
| 6% | dsRNA bands |  |  | Japan, *Castanea* spp. (mostly *C. crenata*), cultured | Peever et al., 1998 |
| 2% | dsRNA bands |  |  | China, *Castanea* spp. (mostly *C. mollissima*), cultured | Peever et al., 1998 |
| 23% | dsRNA bands | 53 different species | No disease (endophytic fungi) | Spain, *Ammophila arenaria*, *Alopecurus arundinaceus*, *Brachypodium sylvaticum*, *Cynodon dactylon*, *Dactylis glomerata*, *Elymus farctus*, *Festuca rubra*, *Holcus lanatus*, and *Lolium perenne*, cultured | Herrero et al., 2009 |
| ~19% | dsRNA bands | *Magnaporthe oryzae* | Rice blast | Vietnam, *Oryza sativa*, field | Urayama et al., 2010 |
| 13% | dsRNA bands | *Aspergillus* | Range of diseases on plants, aspergillosis in vertebrates | North America, not stated, cultured | Elias and Cotty, 1996 |
| 10% | dsRNA bands | *Aspergillus* section *flavi* |  | Worldwide, not stated, cultured | van Diepeningen et al., 2006 |
| 10% | dsRNA bands | *Fusarium oxysporum* | Fusarium wilt | United States, *Glycine max* and diseased soil, cultured (recent ex-field) | Kilic and Griffin, 1998 |
| 4% | dsRNA bands | *Heterobasidion annosum* | Annosus root rot | Various countries including Sweden, Norway, Switzerland, Lithuania and Russia, decayed wood, cultured | Ihrmark et al. 2001 |
| 2% | dsRNA bands | *Fusarium graminearum* | Fusarium head blight | Korea, *Hordeum vulgare* and *G. max*, cultured (recent ex-field) | Chu et al., 2004 |
| 2% | dsRNA isolation and high throughput sequencing | *Fusarium* spp | Fusariosis | Argentina, *G. max* and *Sorghum bicolor*, cultured (recent ex-field) | Jacquat et al., 2020 |
| 0% | dsRNA bands | *Leptosphaeria maculans* | Phoma stem canker | China, *Brassica napus*, field | Shah et al., 2020 |

# References

Arakawa, M., Nakamura, H., Uetake, Y., and Matsumoto, N. (2002). Presence and distribution of Double-stranded RNA elements in the white root rot fungus *Rosellinia necatrix*. *Mycoscience*. 43:1, 21–26. doi.org/10.1007/s102670200004

Bottacin, A. M., Levesque, C. A., and Punja, Z. K. (1994). Characterization of dsRNA in *Chalara elegans.* Phytopathology, 84, 303–312. https://doi.org/10.1094/Phyto-84-303

Chu, Y.-M., Lim, W.-S., Yea, S.-J., Cho, J.-D., Lee, Y.-W., and Kim, K.-H. (2004). Complexity of dsRNA mycovirus isolated from *Fusarium graminearum*. Virus Genes, 28:1, 135–143. https://doi.org/10.1023/B:VIRU.0000012270.67302.35

Elias, K. S., and Cotty, P. J. (1996). Incidence and stability of infection by double-stranded RNA genetic elements in *Aspergillus* section *flavi* and effects on aflatoxigenicity. Canadian Journal of Botany, 74:5, 716–725.

Herrero, N., Sánchez Márquez, S., and Zabalgogeazcoa, I. (2009). Mycoviruses are common among different species of endophytic fungi of grasses. *Archives of Virology*. 154:2, 327–330. doi.org/10.1007/s00705-008-0293-5

Howitt, R. L. J., Beever, R. E., Pearson, M. N., and Forster, R. L. S. (1995). Presence of double-stranded RNA and virus-like particles in Botrytis cinerea. Mycological Res. 99:12, 1472–1478. doi: 10.1016/S0953-7562(09)80795-8

Ihrmark, K., Zheng, J., Stenström, E., and Stenlid, J. (2001). Presence of double-stranded RNA in *Heterobasidion annosum*. Forest Pathology, 31(6), 387–394. https://doi.org/https://doi.org/10.1046/j.1439-0329.2001.00263.x

Ikeda, K., Nakamura, H., Arakawa, M., and Matsumoto, N. (2004). Diversity and vertical transmission of double-stranded RNA elements in root rot pathogens of trees, *Helicobasidium mompa* and *Rosellinia necatrix*. Mycological Research, 108:6, 626–634. https://doi.org/10.1017/s0953756204000061

Jacquat, A. G., Theumer, M. G., Cañizares, M. C., Debat, H. J., Iglesias, J., García Pedrajas, M. D., and Dambolena, J. S. (2020). A survey of mycoviral infection in *Fusarium* spp. isolated from maize and sorghum in Argentina identifies the first mycovirus from *Fusarium verticillioides*. *Viruses.* 12:10, 1161. doi.org/10.3390/v12101161

Khalifa, M. E., and MacDiarmid, R. M. (2021). A mechanically transmitted DNA mycovirus is targeted by the defence machinery of its host, *Botrytis cinerea*. *Viruses*. 13:7, 1315. doi.org/10.3390/v13071315

Kilic, O., and Griffin, G. J. (1998). Effect of dsRNA-containing and dsRNA-free hypovirulent isolates of *Fusarium oxysporum* on severity of Fusarium seedling disease of soybean in naturally infested soil. Plant and Soil, 201:1, 125–135. https://doi.org/10.1023/A:1004319614390

Park, S.-M., Kim, J.-M., Chung, H.-J., Lim, J.-Y., Kwon, B.-R., Lim, J.-G., Kim, J.-A., Kim, M.-J., Cha, B.-J., and Lee, S.-H. (2008). Occurrence of diverse dsRNA in a Korean population of the chestnut blight fungus, *Cryphonectria parasitica*. Mycological Research, 112:10, 1220–1226.

Pearson, M. N., and Bailey, A. M. (2013). Viruses of Botrytis. Adv. Virus Res. Elsevier Inc. 86, 249-272 doi: 10.1016/B978-0-12-394315-6.00009-X

Peever, T. L., Liu, Y.-C., and Milgroom, M. G. (1997). Diversity of hypoviruses and other double-stranded RNAs in *Cryphonectria parasitica* in North America. *Phytopathology*. 87:10, 1026–1033.

Peever, T. L., Liu, Y.-C., Wang, K., Hillman, B. I., Foglia, R., and Milgroom, M. G. (1998). Incidence and diversity of double-stranded RNAs occurring in the chestnut blight fungus, *Cryphonectria parasitica*, in China and Japan. *Phytopathology*, 88:8, 811–817.

Pi-Fang, T., Pearson, M. N., and Beever, R. E. (2004). Mycoviruses in *Monilinia fructicola*. *Mycological Research*, 108:8, 907–912.

Ruiz-Padilla, A., Rodríguez-Romero, J., Gómez-Cid, I., Pacifico, D., and Ayllón, M. A. (2021). Novel mycoviruses discovered in the mycovirome of a necrotrophic fungus. *Mbio*. 12:3, e 03705-20. doi.org/10.1128/mBio.03705-20

Shah, U. A., Kotta-Loizou, I., Fitt, B. D. L., and Coutts, R. H. A. (2020). Mycovirus-induced hypervirulence of *Leptosphaeria biglobosa* enhances systemic acquired resistance to *Leptosphaeria maculans* in *Brassica napus*. *Molecular Plant Microbe Interactions*. 33:1, 98–107. doi.org/10.1094/MPMI-09-19-0254-R

Urayama, S., Kato, S., Suzuki, Y., Aoki, N., Le, M. T., Arie, T., ... & Moriyama, H. (2010). Mycoviruses related to chrysovirus affect vegetative growth in the rice blast fungus *Magnaporthe oryzae.* *Journal of General Virology*, 91(12), 3085-3094.

van Diepeningen, A. D., Debets, A. J. M., and Hoekstra, R. F. (2006). Dynamics of dsRNA mycoviruses in black *Aspergillus* populations. *Fungal Genetics and Biology*, 43:6, 446–452.

Voth, P. D., Mairura, L., Lockhart, B. E., and May, G. (2006). Phylogeography of *Ustilago maydis* virus H1 in the USA and Mexico. *Journal of General Virology*. 87:11, 3433–3441

Telengech, P., Hisano, S., Mugambi, C., Hyodo, K., Arjona-López, J. M., López-Herrera, C. J., Kanematsu, S., Kondo, H., and Suzuki, N. (2020). Diverse partitiviruses from the phytopathogenic fungus, *Rosellinia necatrix*. *Frontiers in Microbiology*. 1064. doi.org/10.3389/fmicb.2020.01064

Yie, S. W., Khalifa, M. E., Hahn, T., and Pearson, M. N. (2014). Molecular characterization of a novel victorivirus from the entomopathogenic fungus *Beauveria bassiana*. *Archives of Virology*. 159:6, 1321–1327. doi.org/10.1007/s00705-013-1938-6
